# Effects of Long-Term Institutionalization on the Linguistic-Communicative Performance of Patients with Schizophrenia

**DOI:** 10.3390/healthcare13202592

**Published:** 2025-10-15

**Authors:** Viviana Vega, Yasna Sandoval, Carlos Rojas, Jaime Crisosto-Alarcón, Ma Gabriela Cabrera, Nicole Almeida, Solange Parra, Gabriel Lagos, Angel Roco-Videla

**Affiliations:** 1 Sanatorio Neuropsiquiátrico y Geriátrico Hualqui Clinic, Concepción, Hualqui 4180000, Chile; vivianavegau@gmail.com; 2 Department of Rehabilitation Health Sciences, University of Bío-Bío, Chillán 3780000, Chile; jcrisosto@ubiobio.cl (J.C.-A.); glagos@ubiobio.cl (G.L.); 3 School of Speech Therapy Faculty of Rehabilitation Sciences and Quality of Life, San Sebastián University, Concepción 4030000, Chile; mgabrielacabreram@gmail.com; 4 Doctorate in Linguistics, Faculty of Humanities and Arts, University of Concepción, Concepción 4030000, Chile; 5 Department of Psychiatry and Mental Health, Faculty of Medicine, University of Concepción, Concepción 4070409, Chile; nalmeidamarcos@gmail.com; 6 Department of Nutrition and Public Health, University of Bío-Bío, Chillán 3780000, Chile; sparra@ubiobio.cl; 7 Vicerrectoría de Investigación e Innovación, Universidad Arturo Prat, Iquique 1110939, Chile; anroco@unap.cl

**Keywords:** schizophrenia, institutionalization, language, communication

## Abstract

**Background/Objectives**: This study examines the impact of long-term institutionalization on the linguistic and communicative abilities of people diagnosed with schizophrenia, focusing on the influence of educational background. Schizophrenia is characterized by cognitive and social deficits, including disruptions to language use and communicative engagement. Prolonged institutionalization can exacerbate these impairments by depriving individuals of essential social interactions and cognitive stimulation. **Methods**: A case series approach was employed with 18 participants, and validated assessment tools such as the Montreal Evaluation of Communication and the Boston Diagnostic Aphasia Test were used to measure communicative performance. **Results:** Participants with higher educational attainment (nine or more years of schooling) who had been institutionalized for ten years or more exhibited significantly better performance than their less-educated counterparts across various communication domains, including comprehension of linguistic prosody, lexical fluency, and auditory comprehension. This implies that completing a higher degree may mitigate the cognitive decline impact of prolonged stays in an institution. However, the study design does not allow us to ascertain whether education functions as a mitigating factor. **Conclusions:** The results highlight the importance of incorporating educational considerations into therapeutic strategies for individuals with schizophrenia, especially those experiencing long-term institutionalization. Providing enhanced educational opportunities within institutional settings could mitigate the adverse effects of prolonged confinement and foster improved communication and social skills. These findings are consistent with research on cognitive reserve, which suggests that education fosters adaptive strategies and the utilization of alternative neural pathways. This enables individuals to maintain communication skills despite the cognitive impairment associated with schizophrenia.

## 1. Introduction

The relationship of schizophrenia with long-term confinement in an institution is an important issue that needs further exploration due to the communicative and linguistic skills in individuals affected by schizophrenia. It is a mental disorder that persists through life and involves a breakdown in the relation between mental and emotional life and between cognition and sociability [1]. Long-term institutionalization is a result of poor mental health and inadequate social support systems, highlighting the range of these disabilities. This situation begs for a methodical assessment of the impact of prolonged institutional confinement on the social functioning of language skills, which is key for interaction and intellectual activity [2,3].

Institutionalization is the term used for the process of placing people with severe physical and mental health issues, such as the elderly or individuals suffering from dementia, into care facilities like nursing homes and mental health centers. This process has a significant impact on a person’s health and wellbeing, including physical and mental health, affecting the overall quality of life [4,5]. Particularly the elderly or people suffering from dementia become more socially withdrawn and dependent on others. Studies have found that the more someone is cared for in an institution, the worse their level of well-being becomes, signaling a problem in the quality of life of the people [4,5].

Recent research has focused on how the institutional context affects the cognitive and communicative health of patients suffering from schizophrenia. Research suggests that patients within institutional settings tend to display a pronounced reduction in emotional engagement and motivation, alongside a combination of communicative deficits, especially in the form of alogia, anhedonia, and attitudinal reduced engagement towards previously enjoyed activities [6]. The character of institutional care tends to lead to a deficit in social interaction, which exacerbates the deficits and curtails the chances to exercise and develop communicative skills, thus choking the potential recovery pathways and rehabilitation efforts [7]. For this reason, studying the impacts of prolonged lockdown on the communication skills of schizophrenia patients is not only important, but also has profound implications for treatment modalities.

Previous findings suggest that educational background plays a key role in such contexts, aiming at the fact that education serves as a cognitive reserve capacity able to provide a barrier against the influences of confinement [8,9]. The relationship between educational achievement and cognitive outcome in schizophrenia shows the possible protective factor provided by education, thus promoting better communicative functions despite the unfavorable conditions associated with long-term care contexts [10,11]. This proposes that together with therapeutic regimens, educational history should be taken into account when planning rehabilitation strategies to foster enhanced language proficiency and social functioning in this population.

The relevance of linguistic deficits in schizophrenia cannot be overstated. Impairments in speech production, comprehension, and participation in conversations are common in these patients, often leading to significant struggles in maintaining social relationships and accessing necessary health services [12]. Moreover, these difficulties in communication are frequently correlated with negative symptoms, including social withdrawal and emotional flatness, which can exacerbate the patient’s overall condition and contribute to a cyclic pattern of disconnection from the social milieu [13,14]. As theories regarding cognitive dysfunction in schizophrenia evolve, there is an imperative to critically analyze how prolonged institutionalization compounds these communicative challenges.

Research indicates that prolonged periods of institutionalization are associated with heightened cognitive impairments, leading to a pattern of deterioration in language skills that could delay recovery prospects [15,16]. Evidence shows that within institutional settings, the lack of engaging and stimulating communicative opportunities can significantly undermine cognitive resources, which are vital for language processing [15,16]. This aspect of care requires urgent reform in institutional practices aimed at enhancing communicative engagement and cognitive stimulation across treatment protocols.

Given the necessity of social interaction when promoting communicative competency, studies highlight the importance of individualized rehabilitation programs, considering the unique experiences of long-term institutionalized patients. Specifically, incorporating cognitive and linguistic training into care contexts within institutional settings can yield promising results in enhancing communicative skills and, thereby, overall quality of life for these individuals [17]. A focus on developing platforms that allow for meaningful interpersonal exchange is essential in any rehabilitative strategy.

In this context, the current study observes the relationships between long-term institutionalization, educational achievement, and communication abilities in individuals diagnosed with schizophrenia. It points out the critical impact of long-term institutionalization on language and communication deficits, since schizophrenia is characterized by disruptions of thought processes, perception, and social functioning. The analysis focuses on how educational background and length of institutionalization influence communication performance in this vulnerable population, which is often stalled in its social reintegration.

The research uses a case series methodology, categorizing participants according to their length of institutionalization and educational background. This approach allows targeted exploration of how educational attainment could lessen cognitive decline and communication deficits resulting from confinement. Prior studies have suggested that a higher level of education can enhance language proficiency in the context of prolonged institutionalization [6,7,8,9]. Findings suggest that understanding these dynamics could inform therapeutic interventions aimed at improving communication skills and facilitating community reintegration for patients exiting long-term care. Additionally, the study’s insights may contribute to the development of evidence-based policies that prioritize cognitive engagement and promote social interaction, ultimately guiding the creation of rehabilitation strategies tailored to the specific needs of this population [18,19].

## 2. Materials and Methods

### 2.1. Participants

This case study involved a total of 18 adult participants (M = 53.83 years; SD = 11.98) who have been diagnosed with schizophrenia who voluntarily agreed to participate in the research. All participants recruited belonged to a long-stay center for people with psychiatric disorders in the city of Concepción, Chile. To investigate the impact of institutionalization on communicative and linguistic abilities, participants were classified into two groups according to the duration of their institutional stays. Group 1 consisted of individuals who had experienced 9 years or fewer in institutional settings, whereas Group 2 comprised those who had been institutionalized for 10 years or more. In Group 1, there were 7 participants (M = 60.14 years; SD = 3.18), who had an average of 8.86 years of schooling (SD = 3.62) and achieved a Mini-Mental Status Examination (MMSE) score of 27.57 (SD = 2.07). In Group 2, 11 participants (M = 49.82 years; SD = 13.86) had a slightly higher average of 9.27 years of schooling (SD = 3.47) and an MMSE score of 26.36 (SD = 1.63). Prior to commencing data collection, all participants were required to provide informed consent, which was duly approved by the ethics committee of the San Juan de Dios Hospital under protocol number 178/2023.

In terms of the institutional environment’s characteristics, all users receive individual psychological and medical care weekly, participate in cognitive training programs three times weekly, and receive speech therapy support weekly. They also engage in daily individual physical activity and group sessions led by caregivers two or three times a week. Finally, users can receive visitors every afternoon.

### 2.2. Eligibility Criteria

Inclusion criteria for this study included: being over 18 years of age and under 65, having a minimum educational background of 6 years of schooling, possessing a medical diagnosis of schizophrenia lasting 6 months or more, and having been institutionalized for at least 6 months. Additionally, participants were required to receive stable pharmacological treatment for at least 6 months, exhibit cognitive self-awareness as per medical verification, and achieve an MMSE score above 23, indicating no significant risk of dementia. Exclusion criteria encompassed individuals with diagnosed neurological comorbidities (such as intellectual disability, mild cognitive impairment, or dementia), or who were unable to complete the communication assessment for any reason.

### 2.3. Materials

The study utilized two key assessment tools: the Montreal Evaluation of Communication [20] (MEC) and the Boston Diagnostic Aphasia Test [21] (abbreviated format, BDAT). The MEC is a culturally sensitive instrument designed to assess communication performance across five key domains. These dimensions include (1) linguistic prosody, which evaluates the individual’s ability to comprehend and produce emotional tone and intonation in speech; (2) lexical–semantic relations, which focuses on the use of appropriate vocabulary; (3) discursive coherence, assessing the fluidity and logical organization of speech; (4) pragmatic abilities, which examines how well individuals engage in and manage social exchanges; and (5) awareness of deficits, which gauges the person’s recognition of their own communicative limitations [4,5]. Specifically, the MEC evaluates how effectively a participant can understand emotional nuances in speech, organize their thoughts semantically, construct coherent narratives, apply pragmatic rules in conversation, and recognize their own communication weaknesses [4,5]. The MEC has been shown to possess strong reliability and validity, with studies illustrating its efficacy across diverse patient populations, including those with traumatic brain injuries and various communicative disorders. Moreover, psychometric validation data for the MEC indicate satisfactory internal consistency and inter-rater reliability, making it a robust tool for assessing communicative competencies in clinical settings (for this study, the version adapted by Cabrera 2021 will be used [22]). Studies have reported Cronbach’s alpha coefficients exceeding 0.80, indicating excellent internal consistency, while clinical validity has been established through correlations with other recognized language assessment tools [1,2,3,23].

The BDAT, another integral component of this research, provides a comprehensive evaluation of language abilities encompassing both oral and written dimensions, including auditory comprehension, repetition, vocabulary retrieval, reading, and writing. The BDAT’s structured assessment format allows clinicians to detect language deficits effectively by comparing individual scores to normative data, thus facilitating the identification of specific areas requiring intervention [8,9]. Psychometric studies of the BDAT indicate solid reliability and validity; for instance, convergent validity has been established through correlations with other standardized assessments such as the Western Aphasia Battery (WAB [22]). The BDAT demonstrates a high inter-rater reliability coefficient (above 0.85) and has been widely used in both clinical and research settings to measure language impairments associated with various conditions, including stroke and neurodegenerative disorders (for this study, the Spanish version will be used). The combination of the MEC and BDAT in this investigation provides a robust framework for assessing the communicative abilities of individuals diagnosed with schizophrenia, offering insights that are crucial for developing effective therapeutic strategies and rehabilitation programs. For further details, see Table 1.

### 2.4. Procedure

Data collection involved administering three individual assessment sessions. The three sessions were held over a period of three weeks, with one session taking place each week. The first two sessions utilized the MEC, each lasting approximately 45 min, followed by a third session in which the Boston Diagnostic Aphasia Test was administered, taking around 30 min [10,11]. All sessions took place in a well-lit and soundproof room to ensure optimized conditions for participation. Assessments were conducted by a certified speech and language pathologist experienced in working with individuals diagnosed with schizophrenia.

Participants were scheduled for assessments in the morning, spaced at least 2–3 h post medication intake, to minimize any pharmacological effects on cognitive performance. Pre-assessment conversations addressed any questions or concerns participants might have had, ensuring clarity in understanding the tasks involved. In cases of interruptions, a standard 5-min break was incorporated before resuming the assessments. Responses were accurately recorded, followed by detailed scoring according to established protocols [12].

### 2.5. Data Analysis

Statistical analyses were conducted to rigorously evaluate the differences in communicative and linguistic performance between the two groups categorized by their duration of institutionalization. To this end, independent samples t-tests were employed to compare the means of various language-related dimensions, such as comprehension of prosody, lexical fluency, and auditory comprehension, while establishing a significance threshold at *p* < 0.05. This statistical approach allows for the determination of whether observed differences in performance between the groups can be regarded as statistically significant or whether they might simply reflect random variations typical of the sample population [3,23].

Descriptive statistics were generated to present an overview of performance metrics across assessment dimensions for each group. This included calculations of means and standard deviations, which provide insights into the average performance and the variability among scores. These metrics facilitate a clearer understanding of how well each group performs across different linguistic tasks, allowing for comparisons that can drive clinical insights and therapeutic strategies [6,7]. Moreover, while the analyses aimed to uncover significant differences, the results indicating the absence of such findings necessitated careful interpretation considering the limited sample size.

### 2.6. Ethical Considerations

All aspects of the study adhered to ethical research standards. Participants provided informed consent after understanding the study’s purpose, procedures, risks, and benefits. The research protocol received ethical clearance from the San Juan de Dios Hospital ethics board (protocol number 178/2023), ensuring protection of participants’ rights and privacy throughout the process. Additionally, measures were implemented to provide psychological support for participants during and after assessments, addressing any emotional discomfort arising from participation.

## 3. Results

This study investigates the impact of long-term institutionalization on the communication and language skills of individuals diagnosed with schizophrenia, with a particular focus on the role of educational background. The findings, summarized in two tables including data from the MEC and BADT, demonstrate the effect of prolonged confinement on key verbal communication skills and comprehension.

Table 2 compares the communication and language skills of patients with schizophrenia who have been institutionalized for fewer than ten years, distinguishing between those with a basic education (eight years or less) and those with a secondary education or higher (nine years or more). While there was a trend towards better scores among participants with higher educational qualifications, none of the results were statistically significant (*p* > 0.05). This suggests that the effect of education on communicative abilities is limited prior to ten years of institutionalization. The lack of significant variance may be due to the small sample size and variability in participants’ linguistic profiles and prior educational experiences.

Table 3 contrasts the performance of individuals who have been institutionalized for over ten years, again segmented by educational level. Significant differences favoring participants with secondary education or higher were observed for measures of comprehension of linguistic prosody, lexical fluency across various intervals, and oral vocabulary, with *p*-values below 0.05. These results suggest that higher educational attainment may protect against the decline of communicative abilities, particularly in chronic institutional environments.

Overall, these findings highlight the importance of considering educational background in the context of long-term institutionalization and its impact on the communication skills of individuals with schizophrenia.

## 4. Discussion

The relationship between long-term institutionalization and linguistic-communicative performance in patients diagnosed with schizophrenia is an important area of research, enabling comprehensive exploration due to its implications on rehabilitation and recovery strategies. Schizophrenia is characterized by multiple cognitive and social deficits, including language use disruptions and communicative engagement [1,2,3,23] Studies have indicated that prolonged institutionalization often deteriorates pre-existing communicative impairments by depriving individuals of essential social interactions and cognitive stimulation. This scenario makes the relationship between cognitive function and communicative capability somewhat complex in vulnerable populations, demonstrating that institutional environments can delay communication skills and overall recovery outcomes in psychiatric patients [6,7].

Research indicates that long-term institutionalization has an adverse effect on the linguistic and communicative performance of individuals with schizophrenia. Participants with a higher level of education often demonstrate relatively well-preserved communication skills, suggesting that a higher level of education may act as a protective factor against the cognitive decline associated with long-term institutionalization [8,9]. These findings align with research indicating that cognitive reserve, bolstered by education, can help mitigate the adverse effects of psychiatric conditions; for instance, Maltais et al. found a correlation between higher educational levels and improved recovery prospects in schizophrenia [10,11]. Therefore, educational attainment appears to be critical in influencing the resilience of communicative skills in the face of long-term isolation, underscoring the necessity for integrative rehabilitation strategies that consider both cognitive and educational dimensions.

The study employed a case series methodology to categorize participants according to the duration of institutionalization and educational background, enabling a focused analysis of linguistic outcomes [23]. Validated assessment tools like the MEC and BDAT enhanced the reliability of measuring communicative performance [24,25]. Statistical analysis revealed no significant differences in the lower institutionalization group; however, meaningful performance gaps were evident for those institutionalized for ten years or more, emphasizing the connection between extended confinement and deteriorating language skills among individuals with less formal education [15,16]. This relationship underscores the need for reform in institutional practices that promote cognitive engagement and communicative interaction.

Findings regarding auditory comprehension and vocabulary usage emphasize the significance of communication skills in individuals with schizophrenia and their overall functioning. Previous studies have suggested that communication difficulties are associated with negative symptoms, such as reduced social interaction and emotional expression [13,14]. Barriers to communication can prevent individuals with schizophrenia from accessing essential health services, leading to significant challenges in social relationships [12]. These findings suggest a strong link between communication impairment and social withdrawal, highlighting the need for rehabilitation efforts focusing on improving communication and facilitating social reintegration [15,16].

Furthermore, the observed differences in communication capabilities related to educational attainment shed light on the cognitive underpinnings of communicative deficits in individuals with schizophrenia. The link between educational history and cognitive performance after institutionalization supports the idea that educational interventions should be a key part of rehabilitation programs designed to improve the social functioning of people with mental health conditions [25]. Recognizing educational background as an influential factor in linguistic proficiency can inform the development of personalized interventions that build on patients’ latent cognitive abilities.

The relationship between educational attainment and cognitive reserve is noteworthy, suggesting that higher educational levels may facilitate greater linguistic fluency despite the obstacles presented by prolonged institutionalization. This finding is consistent with cognitive theories proposing that education enhances the ability to utilize cognitive resources adaptively, thereby supporting language processing and communicative engagement [8,9]. Therefore, incorporating educational programs into therapeutic frameworks could promote cognitive development that offsets the losses experienced due to long-term institutionalization [18].

In a broader context, these findings align with research on communicative and pragmatic disorders in schizophrenia, illustrating the multidimensional impairments that affect functional outcomes [1,2,3,7,8]. This study highlights the importance of rehabilitation approaches that specifically target combinatorial communication strategies, integrating cognitive and social aspects to optimize interventions. Enhanced cognitive remediation strategies should be combined with educational interventions to improve long-term communicative outcomes.

These findings advocate systemic changes to institutional practices, prioritizing cognitive stimulation and active social engagement over passive care approaches. Evidence-based policy initiatives should encourage environments that support rehabilitative strategies and emphasize the importance of cognitive and linguistic training in institutional settings [15,16]. Such initiatives require collaboration between clinicians, educators, and policymakers to redefine care standards for individuals with mental health issues, particularly those with schizophrenia and those affected by long-term institutionalization. Overall, these findings reinforce existing literature on the relationship between long-term institutionalization, educational attainment, and communication skills, offering insights to improve rehabilitation strategies and patient engagement in recovery pathways. Incorporating educational and cognitive factors into treatment protocols for patients in institutions may help to bridge gaps in communicative performance and significantly improve quality of life for people with schizophrenia.

In general, this study uses the cognitive reserve theory as its primary interpretative framework [26]. According to this theory, a higher level of education can offset the negative effects of factors such as prolonged institutionalization on cognitive and communicative performance [27]. However, the findings reveal nuances that are not entirely evident from this theory alone. Firstly, the differential effect of education is only observed after ten or more years of institutionalization. This suggests the existence of a temporal threshold beyond which cognitive reserve becomes critical for maintaining linguistic competencies. This temporal pattern is not an explicit prediction of the theory, indicating a dynamic interaction between the duration of isolation, environmental stimulation, and prior cognitive resources. Secondly, the magnitude of the differences observed in specific tasks, such as prosody and timed verbal fluency, shows that not all linguistic domains benefit equally from education. This implies that certain subprocesses, particularly those related to lexical access speed and prosodic interpretation, depend more on acquired reserve. Together, these findings imply that education does not merely act as a general buffer; rather, it selectively modulates the resilience of specific language components under conditions of chronic social deprivation.

On the other hand, the study shows that individuals with a higher level of education who have been institutionalized for over ten years demonstrate better linguistic performance. However, this interpretation carries the risk of circular reasoning. Education may not be a protective factor in itself but rather a reflection of premorbid linguistic and cognitive abilities that facilitated the attainment of additional years of schooling and superior performance on tests sensitive to educational level. This potential selection bias is not accounted for in the cross-sectional study design with a small sample size, where years of schooling act as a proxy for prior cognitive reserve. Furthermore, the duration of institutionalization and other unadjusted factors, such as age, negative symptoms, and duration of illness, may act as confounding variables, leading to spurious associations.

Finally, the consistency of the findings is also limited by the application of parametric tests with small sample sizes and by the absence of normative scores, which would mitigate the influence of educational background. Therefore, rather than confirming a protective causal effect, the results should be interpreted with caution and complemented by analyses that minimize the educational burden of the tests. In the future, longitudinal studies or randomized controlled cohorts (with larger sample) that differentiate between genuine protective effects and pre-existing differences in linguistic capability are needed.

## 5. Conclusions

This study reveals that long-term institutionalization significantly impairs the linguistic and communicative performance of individuals with schizophrenia. This impairment is particularly evident in their comprehension of prosodic linguistics, lexical fluency, auditory comprehension, and oral expression. However, educational attainment serves as a protective factor against these detrimental effects: participants with nine or more years of schooling demonstrated significantly better performance across various language-related dimensions than those with eight years or less. These findings suggest that educational background influences communication capabilities in individuals with schizophrenia and imply that educational interventions could enhance cognitive skills and social functioning in rehabilitation programs. Consequently, the study emphasizes the need to reform institutional practices to create environments that support cognitive engagement and social interaction.

## Figures and Tables

**Table 1:** General description of the dimensions analyzed (MEC/BDAT).

Item (MEC/BDAT)	Description
Comprehension of Prosodic Lexical Fluency 0–30 s (MEC)	This involves evaluating how well a participant can process the emotional tones and rhythms of language in a brief response window. Understanding prosody is crucial in everyday communication as it aids in interpreting meaning beyond the literal words spoken.
Lexical Fluency 30–120 s (MEC)	The lexical fluency tasks, which are divided into segments of 30 to 120 s, assess the participant’s ability to quickly generate words within a specified category or beginning with a particular letter, like/p/. Fluency is indicative of cognitive functioning and linguistic capabilities; thus, a score in these areas provides insight into the individual’s language processing efficiency.
Auditory Comprehension (BDAT)	This aspect evaluates the ability to understand spoken language, which is critical for effective communication and is often tested with commands or complex materials in the BDAT, helping to identify the severity and nature of the aphasia.
Oral Expression and Vocabulary (BDAT)	Here, the focus is on assessing the ability to express thoughts verbally and the range of vocabulary used. These tests are integral in understanding the productive language skills of individuals with aphasia and can aid in determining appropriate therapeutic interventions.

**Table 2:** Comparison of communication performance in individuals with 10 or fewer years of institutionalization based on educational level.

Dimension	Basic Education (8 Years or Less) n = 5		Secondary Education (9 or More Years) n = 6		*p*-Value *
	Average Score (M)	Standard Deviation (SD)	Average Score (M)	Standard Deviation (SD)	
Comprehension of Prosodic	1.200	0.837	1.000	1.265	0.634
Lexical Fluency (0–30 seg)	6.200	6.210	6.500	6.504	1.000
Lexical Fluency (30–60 seg)	3.400	3.975	5.670	2.066	0.309
Lexical Fluency (60–90 seg)	3.800	4.087	5.330	4.457	0.579
Lexical Fluency (90–120 seg)	4.200	4.266	6.500	3.082	0.405
Total Lexical Fluency	18.000	17.015	24.500	15.605	0.521
Lexical Fluency/p/(0–30 seg)	5.200	2.588	5.000	3.742	0.777
Lexical Fluency/p/total 2 min	12.800	5.404	9.830	7.468	0.463
Auditory Comprehension	2.750	1.708	2.860	2.268	0.781
Oral Expression and Vocabulary	9.750	1.258	10.710	5.707	0.711

* *t*-test (independent sample).

**Table 3:** Comparison of communication performance in individuals with 10 or more years of institutionalization based on educational level.

Dimension	Basic Education (8 Years or Less) n = 4		Secondary Education (9 or More Years) n = 3		*p*-Value *
	Average Score (M)	Standard Deviation (SD)	Average Score (M)	Standard Deviation (SD)	
Comprehension of Prosodic	0.750	0.957	3.330	1.155	0.046 *
Lexical Fluency (0–30 seg)	3.250	2.217	11.000	2.646	0.032 *
Lexical Fluency (30–60 seg)	0.750	0.957	6.330	4.042	0.048 *
Lexical Fluency (60–90 seg)	0.500	1.000	8.670	4.933	0.028 *
Lexical Fluency (90–120 seg)	1.250	1.500	6.000	2.646	0.032 *
Total Lexical Fluency	5.750	4.349	32.000	11.532	0.034 *
Lexical Fluency/p/(0–30 seg)	2.250	2.872	7.670	2.082	0.048 *
Lexical Fluency/p/total 2 min	4.750	4.646	13.330	2.309	0.032 *
Auditory Comprehension	1.750	0.957	4.330	1.528	0.048 *
Oral Expression and Vocabulary	8.250	2.872	14.000	1.000	0.032 *

* *t*-test (independent sample); * *p* > 0.05.

## Data Availability

The raw data supporting the conclusions of this article will be made available by the authors on request.
